# Recombinant antigen-based antibody assays for the diagnosis and surveillance of lymphatic filariasis – a multicenter trial

**DOI:** 10.1186/1475-2883-3-9

**Published:** 2004-09-03

**Authors:** Patrick J Lammie, Gary Weil, Rahmah Noordin, Perumal Kaliraj, Cathy Steel, David Goodman, Vijaya B Lakshmikanthan, Eric Ottesen

**Affiliations:** 1 Division of Parasitic Diseases, Centers for Disease Control and Prevention, MS-F13, 4770 Buford Highway, Atlanta, Georgia, 30341, USA; 2 Infectious Diseases Division, Campus Box 8051, Washington University School of Medicine, 660 S. Euclid Avenue, St. Louis, MO 63110, USA; 3 Institute for Research in Molecular Medicine and School of Medical Sciences, Universiti Sains Malaysia, Malaysia; 4 Centre for Biotechnology, Anna University, Chennai – 600 025, India; 5 Laboratory of Parasitic Diseases, National Institute of Allergy and Infectious Diseases, National Institutes of Health, Bethesda, Maryland, 20892, USA; 6 Emory University Lymphatic Filariasis Support Center, 1518 Clifton Road, Atlanta, Georgia, 30322, USA

## Abstract

The development of antifilarial antibody responses is a characteristic feature of infection with filarial parasites. It should be possible to exploit this fact to develop tools to monitor the progress of the global program to eliminate lymphatic filariasis (LF); however, assays based on parasite extracts suffer from a number of limitations, including the paucity of parasite material, the difficulty of assay standardization and problems with assay specificity. In principle, assays based on recombinant filarial antigens should address these limitations and provide useful tools for diagnosis and surveillance of LF. The present multicenter study was designed to compare the performance of antibody assays for filariasis based on recombinant antigens Bm14, WbSXP, and BmR1. Coded serum specimens were distributed to five participating laboratories where assays for each antigen were conducted in parallel. Assays based on Bm14, WbSXP, or BmR1 demonstrated good sensitivity (>90%) for field use and none of the assays demonstrated reactivity with specimens from persons with non-filarial helminth infections. Limitations of the assays are discussed. Well-designed field studies are now needed to assess sampling methodology and the application of antibody testing to the monitoring and surveillance of LF elimination programs.

## Background

The exponential growth of the lymphatic filariasis elimination program has highlighted the need for tools that can be used to monitor progress toward programmatic endpoints (e.g. when to stop mass treatment) as well as to conduct surveillance to detect any potential resumption of transmission. Measurement of microfilaremia, the recognized gold standard for demonstrating the impact of community-wide interventions, is not an optimal monitoring or surveillance tool because of the requirement for nocturnal blood collection in much of the world and because it is a relatively insensitive test for infection [1]. For *Wuchereria bancrofti*, assessment of antigenemia offers the convenience of daytime testing and greater sensitivity than testing for microfilaremia; however, both microfilaremia and antigenemia develop from months to years after exposure, reducing their utility for detection of low levels of infection or recrudescence of transmission [2-6]. Entomologic methods can be used to monitor filarial infection in mosquitoes and to provide point estimates of transmission intensity; however, as infection levels decline, it may become more difficult to collect sufficient numbers of mosquitoes to demonstrate with confidence that infection is absent [7]. By providing a cumulative measure of exposure to filarial infection, antibody assays may circumvent many of the limitations of methods based on direct detection of the parasite, its antigens or its DNA.

Antibody detection has served as the basis for diagnostic assays for filariasis for many decades. The best of these assays are sensitive for infection but are not specific, both because they cannot distinguish current infection from past infection or exposure to the parasite and because there is some degree of cross-reactivity with other helminth infections. On the positive side, prior studies have shown that virtually all residents of filariasis-endemic areas mount antifilarial antibody responses within the first few years of life. Thus, prevalence rates of antifilarial antibodies in children may be a useful index for assessing changes in transmission of the infection [8,9].

Antibody assays based on crude filarial extracts are limited by cross-reactions with other nematode antigens [10]. Recombinant filarial antigens should, in principle, be more useful as the basis of diagnostic or exposure assays because of their greater specificity. As a first step in the development and validation of such assays, we conducted a multicenter evaluation of antibody-based diagnostic assays using 3 recombinant antigens, Bm14, WbSXP and BmR1.

Bm14 and WbSXP belong to a family of related genes that encode proteins that are strong immunogens in many parasitic nematode infections [11]. SXP and Bm14 were originally isolated from cDNA libraries based on their strong recognition by antibodies from microfilaremic persons, and both have been developed as candidates for diagnostic assays [12,13]. Assays based on detection of IgG4 antibodies to Bm14 have sensitivities of 85–90% when serum specimens from microfilaremic persons are tested [14,15]. This antigen is reported to be equally sensitive for sera from patients infected with *Brugia malayi *or *Wuchereria bancrofti*. Comparable results have been reported with assays for SXP [11,16,17]. BmR1 encodes a secreted antigen selected from a *B. malayi *cDNA library by antibody screening [18,19]. ELISA and dipstick formats of BmR1 assays have been reported to have a sensitivity of >90% when serum specimens from persons with *B. malayi *or *B. timori *microfilaremia were tested [18-22].

The present study was designed to compare objectively the performance of antibody assays for filariasis based on recombinant antigens Bm14, WbSXP, and BmR1. We report here the results of this multicenter evaluation.

## Materials and Methods

### Serum Specimens

Serum specimens from patients of known infection status (see Table 1) were sent to the U.S. Centers for Disease Control and Prevention (CDC). For persons with filariasis, infection was diagnosed by detection of microfilariae. Each specimen was assigned a code number and aliquotted into 5 tubes (100 – 200 μl per tube). A panel of coded serum samples was sent to each of the five participating laboratories along with antigens and assay kits for testing. Results were sent back to CDC for data analysis.

**Table 1:** Sensitivity and Specificity of Antibody Assays^1^

Infection	Bm14 ELISA Positive/Tested (%)	SXP Cassette Positive/Tested (%)	BmR1 ELISA Positive/Tested (%)	BmR1 Dipstick Positive/Tested (%)
W. bancrofti (n = 35)^2^	32/35 (91%)	30/33 (2 NC) (91%)	14/31 (4 NC) (45%)	17/30 (5NC) (56.7%)
B. malayi (n = 28)^3^	27/28 (96%)	7/18 (10 NC) (39%)	28/28 (100%)	27/27 (1NC) (100%)
O. volvulus (n = 20)^4^	11/16 (4 NC) (69%)	9/15 (5 NC) (60%)	0/20 (0%)	1/20 (5%)
Loa (n = 10)^5^	7/9 (1 NC) (78%)	3/7 (3 NC) (43%)	0/10 (0%)	0/9 (1 NC) (0%)
Other (incl. S*trongyloides, Echinococcus*; n = 20)^6^	0/19 (1 NC) (0%)	0/19 (1 NC) (0%)	0/20 (0%)	0/20 (0%)

^1^Specimens were collected from persons with documented infections with the listed parasites; for patients with filarial infections, microfilariae were detected. Abbreviations: NC, no consensus; specimens with a no consensus result were not included in the denominators for calculations.

^2^Geographic source of specimens provided in Table 2.

^3^Geographic source of specimens provided in Table 2.

^4^Ten specimens were from Guatemala and 10 were from Ecuador.

^5^Specimens were collected from patients in Benin.

^6^*Echinococcus *specimens were collected in Kenya; *Strongyloides *specimens were from several settings where lymphatic filariasis was not endemic.

### Assay formats

The Bm14 and BmR1 tests included in the evaluation are based on the detection of antifilarial IgG4 antibodies. The Bm14 assays were performed according to procedures described by Weil et al. [15]. Three different assay formats were used to test for BmR1: an ELISA [18], a dipstick [20] and cassette. All were produced by Malaysian Bio-Diagnostics, Research, Sdn, Bhd. A rapid cassette test for IgG antibody to WbSXP was produced by Span Diagnostics, Ltd (Sachin, India). All participating labs followed assay instructions provided by the assay or kit supplier.

### Analysis of Results

Results of ELISA assays were determined using cut offs defined by the assay developer. For qualitative tests, each laboratory determined whether the appropriate band or spot was visible. To collate results for a given assay with a specific serum sample, a consensus result (either positive or negative) was defined on the basis of agreement among at least 4 of 5 labs. If only 3 of 5 labs obtained the same result or if 3 did and one of the two remaining laboratories did not obtain an interpretable result, this was considered to represent a lack of consensus (recorded as 'NC' or no consensus in Tables 1 and 2). Only two labs used the BmR1 cassette to test the specimens. Achieving consensus required two identical results for this test. Inter-laboratory agreement was assessed with Kappa coefficients, a measure of pair-wise agreement among observers making categorical judgments. For Bm14 and BmR1 ELISA, categorical assignments of positive or negative results were based on criteria established by the test developers.

**Table 2:** Regional Differences in Antigen Recognition

Infection Location (serum source)	Bm14 ELISA	SXP Cassette	BmR1 ELISA
W. bancrofti			
Cook Is.	10/10	9/9 (1 NC)	4/8 (2 NC)
PNG	9/10	9/9 (1 NC)	6/10
India	9/10	9/10	4/8 (2 NC)
Kenya	2/3	1/3	0/3
Haiti	2/2	2/2	0/2
B. malayi			
Indonesia	10/10	0/5 (5 NC)	10/10
India	7/7	4/6 (1NC)	7/7
Malaysia	10/11	3/7 (4 NC)	11/11

## Results

The Bm14 ELISA displayed comparable sensitivity for both *W. bancrofti *(91%) and Brugia (96%) infections, while the other two tests performed better with specimens from the homologous infections (Table 1). For example, the BmR1 ELISA was positive for 100% of the samples from *Brugia *patients, but displayed only modest sensitivity (45%) in terms of its performance with *W. bancrofti *samples. Results were comparable for both the BmR1 dipstick and cassette (data not shown). Similarly, the WbSXP assay was positive for 30 of 33 (91%) serum specimens from *W. bancrofti *patients, but only 39% of *Brugia *cases.

BmR1 assays were remarkably specific for *Wuchereria *and *Brugia *infections, and there was little reactivity with specimens from persons with *O. volvulus*, *Loa loa *or other helminths (e.g. *Strongyloides*)*. *The Bm14 assay, and to a lesser extent, the WbSXP assay, appeared to function as a 'pan-filaria' assay, showing reactivity with the specimens from persons with *W. bancrofti*, *B. malayi*, *L. loa *and *O. volvulus*. None of the assays demonstrated reactivity with specimens from persons with non-filarial helminth infections (Table 1) or with hyper-IgE syndrome (data not shown).

When the geographic source of the serum specimens was considered, additional heterogeneity in responsiveness was noted. For example, although only a limited number of specimens were available for testing, 4 of 6 serum specimens from persons from India infected with *B. malayi *were positive using the WbSXP cassette; however, none of those from Indonesia were positive with this assay (Table 2).

Inter-laboratory categorical agreement for the ELISA assays was quite good (Table 3). Rapid format tests, though convenient, often presented problems of interpretation, independent of the test. Some labs reported the presence of weak bands or dots with control sera. This resulted in a significant number of 'no consensus' results (Table 1) as well as the lower kappa scores associated with the rapid tests (Table 3).

**Table 3:** Inter-lab Agreement for the Different Diagnostic Tests

Assay	Range of Kappa statistics	Mean
Bm14 ELISA	0.690 – 1.00	0.88
SXP Cassette	0.612 – 0.912	0.73
BmR1 ELISA	0.878 – 0.982	0.93
BmR1 Dipstick	0.817 – 0.965	0.87
BmR1 Cassette	0.546 – 1.00	0.80

Kappa statistics were derived from 10 pair-wise inter-lab comparisons.

## Discussion

Assays based on Bm14, WbSXP, or BmR1 demonstrate adequate sensitivity for field use. The Bm14 assay appeared to function as a 'pan-filaria' assay, demonstrating antibody reactivity in the sera from patients with *W. bancrofti*, *B. malayi*, *L. loa *and *O. volvulus*. Although this cross reactivity makes the Bm14 assays useful for monitoring either bancroftian or brugian filariasis, cross reactivity with *O. volvulus *and *L. loa *may limit its utility in some areas of sub-Saharan Africa. The BmR1 assays were sensitive for *B. malayi *infection but relatively insensitive for *W. bancrofti *infection. They showed excellent specificity for *Brugia *and *Wuchereria*, with little reactivity with sera from persons infected with other parasites, including *L. loa *and *O. volvulus*. These results suggest that it may be useful to study the *W. bancrofti *homologue of BmR1 to determine if it is as specific for *W. bancrofti *as BmR1 is for *Brugia*. Unfortunately, recent work suggests that this is not the case [23].

For mapping the distribution of lymphatic filariasis, rapid antibody tests may provide acceptable sensitivity, depending on the geographic area where the mapping is to be done, but the potential for problems with specificity (both in distinguishing past exposure from present infection as well as differentiating filarial from non-filarial infection) still remains. For mapping *W. bancrofti*, there is minimal value in using antibody tests instead of the antigen tests that are currently used, but because there is no antigen test for *Brugia*, antibody tests might be an alternative for mapping the distribution of these infections. In *Brugia*-endemic areas, it will be important to demonstrate, however, the relationship between prevalence rates for microfilaremia and antibodies to validate the assay as a useful tool for programmatic decisions. At this point, it is not clear what antibody prevalence should be considered an indication to initiate mass treatment; no attempt was made in this study to distinguish between antibody responses associated with active infection and those triggered by exposure [24,25].

Antibody assays almost certainly will find other uses in the context of lymphatic filariasis (LF) elimination programs. For example, antifilarial antibody tests may be sensitive markers of transmission intensity or provide evidence of ongoing exposure to filarial infection long before the development of antigenemia or microfilaremia. Primates develop antibody responses to Bm14 within 4–8 weeks following *B. malayi *infection [26]. In a longitudinal study in Egypt, microfilaria-negative persons who were positive for Bm14 antibody at baseline were more likely to be microfilaria-positive after one year than were Bm14-negative persons [15]. Less is known about the kinetics of antibody responses to BmR1. Since antibody responses provide an early indicator of infection, assays for antifilarial antibodies should be useful for surveillance following initiation of LF elimination programs.

As LF programs reach their planned end point (5 or more years of > 80% drug coverage in targeted populations), it will be necessary to determine whether or not transmission has been interrupted and whether mass drug administration can be stopped. Parasitologic testing, whether for microfilaremia or antigenemia, will require testing of thousands of persons to demonstrate that infection levels are below 0.1%, the level established by the Global Program as the end point for defining the elimination of LF. Since antibody responses develop in the absence of demonstrable infection, detecting incident antibody responses should provide a more sensitive measure of transmission than microfilaria or antigen detection. Children born following the cessation of transmission should be antibody-negative, while older children and adults may have evidence of residual antibody reactivity [8,9,27]. A testing strategy based on screening of children could be exploited for ongoing surveillance in the aftermath of LF programs and may not require screening of as many children as called for by current testing guidelines. The absence of antibody in appropriately chosen populations would strongly suggest that transmission has been interrupted. Additional studies are needed to test the value of antibody testing as a tool for certifying the elimination of filariasis transmission.

Operational use of antibody assays requires far more practical experience with the assays than we now have. Of greatest concern is the specificity of the tests employed. For example, ELISA tests often use a statistical approach to establish cutoff values for positive results. A test that is 99% specific will predictably have some false positive results if large numbers of samples are tested. Further work will be needed to establish rates of antibody positivity that exceed the number that are likely to occur by chance. In addition, it will be necessary to develop and validate algorithms for confirming the presence of infection or ongoing transmission in situations where low antibody prevalence rates are detected. Despite these caveats, we believe that antibody tests based on antigens like Bm14, BmR1, and Wb-SXP will prove to be useful tools that can be used to facilitate decision making by program managers in the context of filariasis elimination programs.

## Competing Interests

GW, RN, and PK have relationships with companies interested in developing commercial applications of the Bm14, BmR1 and WbSXP assays, respectively.

## Authors' Contributions

GW, RN, PK, and VBL were responsible for the initial development of the assays. All of the authors participated in the planning of this multicenter study. DG was responsible for coordinating specimen shipment and database management. Participating labs included PL and DG from CDC, RN from the Universiti Sains Malaysia, PK and VBL from Anna Center for Biotechnology, CS from NIH and GW from Washington University School of Medicine. PL and EO were responsible for coordinating the study. PL wrote the first draft of the manuscript, but all of the authors participated in the editing of subsequent versions.
